# Effect of Print Orientation and Post-Curing Duration on the Flexural Strength, Flexural Modulus and Vickers Microhardness of a 3D-Printed Occlusal Splint Resin

**DOI:** 10.3390/bioengineering12121322

**Published:** 2025-12-04

**Authors:** Mustafa Sahin, Suleyman Kutalmış Buyuk, Huseyin Simsek, Tuncer Akdoğan, Safiyenur Bircan, Mustafa Serdar Toroglu

**Affiliations:** 1 Department of Orthodontics, Faculty of Dentistry, Çukurova University, Adana 01250, Türkiye; mustafasahin.93@hotmail.com (M.S.); storoğlu@cu.edu.tr (M.S.T.); 2 Department of Orthodontics, Faculty of Dentistry, Ordu University, Ordu 52200, Türkiye; 3 Department of Pedodontics, Faculty of Dentistry, Ordu University, Ordu 52200, Türkiye; dr.huseyinsimsek@gmail.com (H.S.); safiyenur.bircan@odu.edu.tr (S.B.); 4 Department of Oral and Maxillofacial Surgery, Faculty of Dentistry, Çukurova University, Adana 01250, Türkiye; takdogan@cu.edu.tr

**Keywords:** 3D printing, occlusal splint, build orientation, post-curing, flexural strength, flexural modulus, Vickers hardness, additive manufacturing

## Abstract

Additive manufacturing (AM) offers precision and efficiency in occlusal splint fabrication; however, the combined influence of build orientation and post-curing duration on the mechanical performance of splint resins remains insufficiently explored. This in vitro experimental study evaluated the effects of three build orientations (0°, 45°, and 90°) and three post-curing protocols (uncured, standard, and extended) on the flexural strength (FS), flexural modulus (FM) and Vickers hardness number (VHN) of a Class IIa biocompatible occlusal splint resin (NextDent Ortho Rigid). A total of 180 specimens were fabricated using a vat polymerization-type 3D printing system. Statistical analyses were conducted using one-way analyses of variance and Tukey’s tests at a significance level of α = 0.05. Both build orientation and post-curing duration significantly affected FS and VHN (*p* < 0.001). The combination of 45° build orientations and extended post-curing produced the highest FS (169.76 MPa) and FM (7502.17 MPa), exceeding values typically reported for 3D-printed splints, while the 90° orientation with extended curing achieved the highest VHN (21.88). Hardness gains, however, plateaued beyond standard curing, indicating a trade-off between strength and surface hardness. These results demonstrate that print orientation and post-curing time are decisive parameters in optimizing the mechanical performance of 3D-printed occlusal splints. For high-load clinical applications such as bruxism, prioritizing flexural strength over surface hardness may improve appliance longevity, supporting 45° orientation with extended curing as an evidence-based manufacturing approach.

## 1. Introduction

Occlusal splints are fundamental appliances in the conservative management of temporomandibular disorders (TMD) and bruxism, designed to reduce hyperactivity of the masticatory muscles, protect dentition from parafunctional forces, and maintain occlusal stability [1,2,3]. Bruxism and temporomandibular disorders are highly prevalent in society, and occlusal splints are among the most frequently used appliances in the management of these conditions. Especially in cases of severe bruxism, maintaining the splint’s long-term durability under high occlusal loads is critical to treatment success. Clinical reports indicate that splint fractures occurring in the posterior region negatively impact patient compliance and create difficulties with treatment adherence. Therefore, determining parameters to enhance the mechanical performance of the materials used is important for both clinicians and patients. Given the multifactorial nature of temporomandibular disorders (TMD), conservative approaches such as occlusal splints remain a primary modality for symptom management [4]. Traditional fabrication workflows, comprising alginate impressions, stone model pouring, wax-up, and manual polymerization are labor-intensive, time-consuming, and prone to operator variability [5,6,7]. These processes can delay delivery, increase laboratory costs, and introduce inaccuracies that compromise fit and comfort.

Advances in digital dentistry, especially the integration of computer-aided design/computer-aided manufacturing (CAD/CAM) and additive manufacturing (AM), have transformed occlusal splint production. Digital workflows allow for direct fabrication from intraoral scan data, bypassing conventional impression and casting steps, thereby reducing turnaround time and enhancing precision [8,9,10]. Among AM technologies, vat photopolymerization enables the layer-by-layer fabrication of complex geometries with high resolution [11,12]. 3D printing has been widely adopted in dentistry for the fabrication of crowns, bridges, surgical guides, and orthodontic models [13], while its application in occlusal splint production has been increasingly explored in recent years [14]. Furthermore, various print orientations have been shown to affect the mechanical properties of such dental devices, contributing to optimization efforts in clinical use [15]. Despite its advantages, AM produces anisotropic structures, wherein mechanical properties depend on the build orientation relative to functional loading [16,17,18]. Although numerous studies have been conducted on 3D-printed dental appliances in recent years, full factorial studies that consider both print orientation and post-curing times are limited. The literature has generally investigated the effects of a single parameter (e.g., only the print angle or only the post-curing time). This makes it difficult to offer clear recommendations that will be reflected in clinical practice. Furthermore, existing findings demonstrate discrepancies between different resin types and printer technologies, necessitating the need for standardized protocols. Print orientation refers to the angulation at which an object is positioned relative to the build platform during AM. This parameter directly affects anisotropy in mechanical properties, interlayer bonding, dimensional accuracy, and surface quality, thereby impacting the functional longevity of the printed device. Previous work has confirmed that both layer thickness and build orientation significantly influence the flexural strength of printed dental resins. Likewise, Castro et al. [19] demonstrated that print orientation alters surface hardness and dimensional accuracy, indicating that no single orientation is universally optimal. Inadequate selection of build orientation can compromise fracture resistance, particularly under parafunctional forces. Moreover, post-curing is essential to increase the degree of monomer conversion, enhance mechanical integrity, and reduce residual monomers, thereby improving biocompatibility. Extended post-curing durations may further enhance cross-link density and surface hardness, though over-curing could potentially lead to embrittlement [20,21,22,23,24,25]. Certainly, extended post-curing can further increase cross-link density; however, beyond approximately 40–60 min, improvements in mechanical properties tend to plateau or even decline [26].

Another important factor is the choice of dental material. Resin-based composites, splint resins, and provisional crown polymers each demonstrate variable mechanical outcomes depending on both material composition and processing parameters. Recent optimization frameworks, such as multi-criteria decision-making models, have been applied to rank restorative composites according to mechanical and clinical performance, emphasizing the importance of parameter selection in dental materials [27].

AM has become increasingly popular in dentistry, particularly for the fabrication of occlusal splints. A recent narrative review underscores the growing adoption of 3D-Printed splints in clinical practice [28]. However, the mechanical performance of printed splint materials still does not match that of conventional approaches. In addition to mechanical outcomes, 3D-Printed provisional restorations have been shown to differ from conventionally fabricated crowns in terms of surface roughness and biofilm adhesion [29], further underlining the clinical relevance of optimizing printing parameters. For example, one study reported flexural strength values of common 3D-printed splint resins ranging from ~50 to 100 MPa, whereas traditional heat-polymerized or milled acrylic splints achieve around 100–110 MPa [30]. Even among conventional polymers, mechanical properties vary substantially; Uçar et al. [31] showed that polyamide resins offer higher flexibility, however lower stiffness compared to polymethyl methacrylate (PMMA), underscoring how composition affects performance. Such disparities emphasize the need to optimize printing parameters to enhance the durability of printed splints [32]. Previous studies have investigated the influence of either print orientation or post-curing protocol in isolation. Diken Turksayar et al. [33] reported that horizontal (0°) orientation yielded the greatest flexural strength in interim crown resins, contrasting with other studies favoring oblique angles. Simeon et al. [23] demonstrated that print orientation significantly alters the flexural properties of printed dental resins, while increasing UV post-curing time generally improves mechanical properties up to a point. However, none of these studies systematically examined the combined influence of orientation and post-curing on splint material properties, and many employed only one-factor-at-a-time experimental designs that limit the analysis of potential interaction effects between these factors. One recent report did evaluate both parameters for a dental 3D-Printed resin, finding that horizontal (0°) orientation yielded the highest flexural strength and that longer post-cure times increased strength further [21]. However, that study focused only on flexural strength (FS) and used specific pre-conditioning (thermal cycling) that differs from the present approach, leaving the combined effect on strength and hardness under standard conditions still unclear. Recent optimization analyses have further demonstrated that print orientation, layer thickness, and post curing time significantly influence the mechanical behavior and sustainability of occlusal splint resins [18].

Therefore, the aim of the present study was to evaluate three clinically relevant build orientations (0°, 45°, 90°) and three post-curing protocols (uncured, standard 10 min, extended 20 min) in a Class IIa biocompatible occlusal splint resin using a 3 × 3 full factorial design under standardized ISO 4049 testing conditions. This approach addresses the existing gap by considering both factors simultaneously. The results of this study are expected to provide evidence-based recommendations for optimal 3D-printing parameters in clinical splint manufacturing. Accordingly, the following null hypotheses were tested: (i) build orientation has no significant effect on FS and Vickers hardness (VHN) of the tested resin, and (ii) post-curing duration has no significant effect on FS and VHN of the tested resin.

## 2. Materials and Methods

This in vitro experimental study employed a 3 × 3 full factorial design to evaluate the independent and combined effects of three build orientations (0°, 45°, and 90°) and three post-curing protocols (uncured, standard, and extended) on the FS and Vickers VHN) of a 3D-printed occlusal splint resin. The orientations were selected based on prior research indicating distinct mechanical behavior due to anisotropy in layer arrangement:

- 0° (horizontal): layers aligned parallel to the load direction, minimizing interlayer stress during bending [26].
- 45° (oblique): stress distributed across multiple layers, potentially optimizing FS [23].
- 90° (vertical): layers stacked perpendicular to the load, resulting in lower flexural strength due to weaker interlayer bonding [34].

The extended curing time (20 min) was specifically selected as a two-fold increase in the standard protocol (10 min). This duration was chosen to ensure that the material reached its maximum potential degree of conversion and to test if the manufacturer’s recommended time was sufficient for optimal mechanical performance, a strategy consistent with previous investigations into polymerization kinetics [21,25,35].

A power analysis (G*Power v3.1, Heinrich Heine University, Düsseldorf, Germany) determined that a minimum of *n* = 9 specimens per subgroup was required to detect a large effect size (f = 0.4) with 80% power at α = 0.05. To ensure adequate statistical robustness, *n* = 10 specimens were prepared for each test condition, resulting in a total of 180 specimens (90 for FS, 90 for VHN).

The specimens were digitally designed using CAD software (TinkerCAD, Autodesk, Montreal, QC, USA) in accordance with ISO 4049 [36] (Dentistry—Base Polymers—Part 2: Orthodontic Base Polymers).

- Flexural strength and modulus specimens: Rectangular bars, 2.0 ± 0.1 mm × 2.0 ± 0.1 mm × 25.0 ± 0.1 mm.
- Vickers hardness specimens: Discs, 10.0 ± 0.1 mm diameter × 2.0 ± 0.1 mm thickness.

Flexural strength specimens were prepared as rectangular bars (2.0 ± 0.1 mm × 2.0 ± 0.1 mm × 25.0 ± 0.1 mm) in accordance with ISO 4049:2019 (Dentistry—Polymer-based restorative materials). These dimensions are widely accepted for flexural testing of polymeric dental resins and are particularly suitable for additive manufacturing, where accurate layer stacking and uniform curing require smaller geometries compatible with the printer’s build platform. Similar dimensions have been adopted in previous studies investigating 3D-printed dental resins and restorative materials, ensuring methodological consistency across research designs [19,21,23]. Vickers hardness specimens were prepared as discs (10.0 ± 0.1 mm diameter × 2.0 ± 0.1 mm thickness).

Printing was performed using an LCD-based vat photopolymerization 3D printer (Phrozen Sonic Mini 8K, Phrozen Inc., Hsinchu, Taiwan) with a 50 μm layer thickness and a pixel resolution of 22 μm. The resin used was NextDent Ortho Rigid (Vertex-Dental BV, Soesterberg, The Netherlands), a Class IIa biocompatible material indicated for occlusal splints.

**Figure 1:**
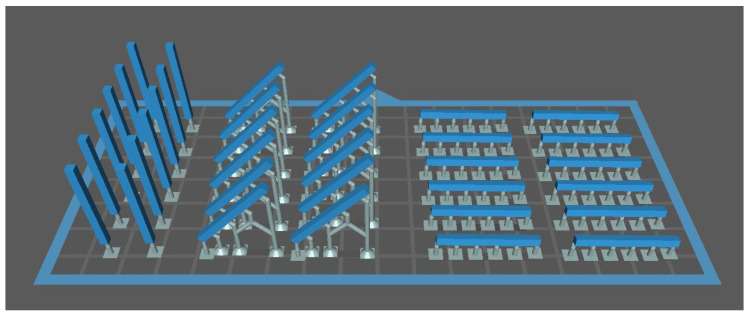
Slicing layout of flexural strength specimens positioned at 0°, 45°, and 90° build orientations.

**Figure 2:**
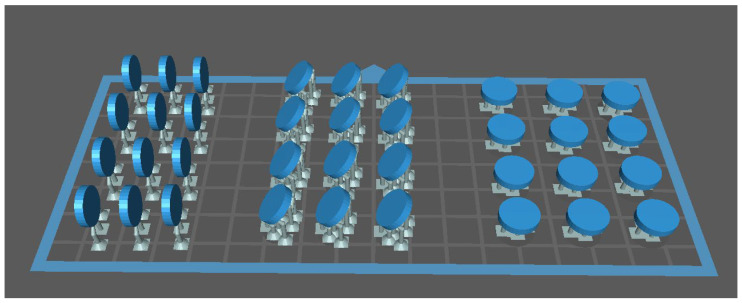
Slicing layout of Vickers hardness specimens arranged at 0°, 45°, and 90° build orientations.

The STL files were oriented in the slicing software (CHITUBOX v1.9, CBD-Tech, Shenzhen, China) according to the assigned build orientation (0°, 45°, or 90°). Printing supports were generated automatically and manually optimized to minimize warpage (Figure 1 and Figure 2).

Immediately after printing, all specimens were rinsed in 99% isopropyl alcohol (IPA) (Merck KGaA, Darmstadt, Germany) for 3 min in an ultrasonic cleaner (Elmasonic S 30H, Elma Schmidbauer GmbH, Singen, Germany) and air-dried with oil-free compressed air. After cleaning, the samples were left at room temperature for 30 min to allow complete solvent evaporation. This procedure is considered critical for removing residual resin from the surface and homogenizing polymerization.

The following post-curing protocols were applied using a curing unit (Phrozen Curing Unit, Phrozen Inc., Hsinchu, Taiwan) equipped with 48 W power (wavelength 405 nm):

- Uncured (Control): no additional polymerization after printing and cleaning.
- Standard curing: 10 min, as recommended.
- Extended curing: 20 min (double the standard time).

Specimens were positioned to ensure uniform light exposure on all surfaces during curing.

Before mechanical testing, all specimens were stored in distilled water at 37 °C for 24 h to stabilize short-term water sorption and to standardize post-polymerization mechanical properties. Prior to testing, specimens were gently dried with absorbent paper to remove any surface moisture. FS was measured according to ISO 4049 in a universal testing machine (AGS-X, Shimadzu Corp., Kyoto, Japan) using a three-point bending setup:

- Support span: 20 mm.
- Crosshead speed: 1 mm/min (ISO-compliant).
- Load applied until fracture.

FS was calculated using the equation:

$$\sigma f=\frac{3FL}{2bh^{2}}$$

where

- *F* = maximum load at fracture (N);
- *L* = support span (mm);
- *b* = specimen width (mm);
- *h* = specimen height (mm).

The flexural modulus (FM) was calculated using the following formula:

$$E=\frac{F_{1}L^{3}}{4bh^{3}d}$$

- *E*: Flexural Modulus (MPa);
- *F*_1_: Load (N)—taken at the point of maximum slope in the linear portion of the load–deflection curve;
- *L*: Span between supports (mm)—typically 20 mm according to ISO 4049;
- *b*: Specimen width (mm);
- *h*: Specimen height (mm);
- *d*: Deflection (mm) at the applied load *F*_1_

VHN was determined using a microhardness tester (FM-700, Future-Tech Corp., Kawasaki, Japan) with the following parameters:

- Load: 500 g (4.9 N).
- Dwell time: 5 s.

Each specimen received three indentations on the polished surface, which had been sequentially finished under running water with 800-, 1200-, and 2000-grit silicon carbide papers and subsequently polished with a 0.05 µm alumina suspension to ensure a standardized surface finish. The indentations were spaced at least 1 mm apart, and the mean VHN was calculated using:

$$VHN=\frac{1.8544\cdot F}{d^{2}}$$

where *F* is the load in kgf and *d* is the mean diagonal length of the indentation in mm.

Specimens were randomly allocated to test sequences within each group using a random number table to minimize allocation bias. In addition, the operator performing the Vickers hardness measurements was blinded to the group assignments to reduce the risk of measurement bias

### Statistical Analysis

Statistical analyses were performed using SPSS Statistics v28.0 (IBM Corp., Armonk, NY, USA). Data normality was assessed using the Shapiro–Wilk test. A one-way analysis of variance was performed to test main and interaction effects on FS, FM and VHN. When significant, one-way analysis of variance followed by Tukey’s HSD was used for multiple comparisons. Significance was set at α = 0.05.

## 3. Results

One-way analysis of variance revealed significant main effects of print orientation and post-curing time on FS and FM (*p* < 0.001), with a significant interaction. Overall, cured specimens exhibited significantly higher FS, FM and VHN values than uncured specimens. Flexural strength increased by approximately 69–150%, whereas VHN improved by 15–28% after post-curing, confirming the decisive influence of additional polymerization on both parameters.

Across all build orientations, uncured specimens exhibited the lowest FS values, ranging from 55.70 MPa (90°) to 88.98 MPa (45°). At 0°, FS increased from 65.06 MPa (uncured) to 149.40 MPa (standard curing) and 152.50 MPa (extended curing), representing increases of 129.6% and 134.4%, respectively, compared to the uncured control. At 45°, FS improved from 88.98 MPa (uncured) to 150.11 MPa (standard) and peaked at 169.76 MPa (extended), corresponding to increases of 68.7% and 90.9% over the control. At 90°, FS rose from 55.70 MPa (uncured) to 128.50 MPa (standard) and 139.00 MPa (extended), representing increases of 130.7% and 149.5%, respectively.

Post-curing had a dominant effect on FS of all cured specimens (128.50–169.76 MPa) significantly outperformed their uncured counterparts (55.70–88.98 MPa, *p* < 0.001). Among cured groups, 45° orientation with extended curing yielded the highest FS, which was significantly greater than all other subgroups (*p* < 0.05, Tukey HSD). In contrast, the 90° orientation consistently produced the lowest FS within each curing protocol (*p* < 0.05) (Table 1).

The highest mean FS was recorded in the 45° extended curing group (169.76 MPa). In contrast, vertically printed (90°) specimens showed lower FS values even after curing, likely due to weaker interlayer bonding when the load was applied perpendicular to the printed layers.

The flexural modulus (FM) values are presented in Table 2. Significant differences were observed across build orientations and post-curing conditions (*p* < 0.001). Extended post-curing generally resulted in higher modulus values compared to uncured and standard-cured groups, indicating greater material stiffness. The highest mean FM was recorded for the 45° orientation under extended curing (7502.17 MPa), whereas the lowest was observed in the 90° orientation without curing (2035.45 MPa). These results follow a similar trend to flexural strength, suggesting that both stiffness and strength are enhanced by optimal layer alignment and prolonged polymerization.

Uncured specimens recorded the lowest VHN values, ranging from 17.07 (90°) to 17.49 (45°). At 0°, VHN increased from 17.39 (uncured) to 22.00 (standard) and slightly decreased to 21.16 (extended); however, this numerical reduction was not statistically significant (*p* > 0.05), as both groups shared the same letter annotation in Table 3. At 45°, VHN rose from 17.49 (uncured) to 20.35 (standard) and 19.37 (extended), representing increases of 16.3% and 10.7%. At 90°, VHN increased from 17.07 (uncured) to 21.40 (standard) and peaked at 21.88 (extended), equivalent to increases of 25.4% and 28.2%.

Post-curing improved hardness across all orientations, with the 90° extended curing subgroup achieving the highest VHN, significantly greater than most other subgroups except 0° standard curing (*p* > 0.05). Interestingly, extended curing did not consistently yield higher VHN than standard curing, suggesting a possible plateau effect in hardness gain (Table 3).

The highest hardness was observed in the 90° extended curing group (21.88 VHN). However, the difference compared with the standard curing protocol was minimal, amounting to only a ~2% increase. This suggests that hardness quickly reaches a plateau, as surface polymerization is completed within shorter exposure times, whereas flexural properties continue to benefit from extended curing.

The results highlighted a trade-off between FS and VHN, 45° extended curing was optimal for FS but not for VHN, 90° extended curing maximized VHN but produced the lowest FS among cured groups.

These findings highlight that FS and VHN do not always increase in parallel. While the 45° extended curing combination provided the highest FS values, the 90° extended curing group showed the highest VHN but the lowest FS. This divergence underscores the need to prioritize flexural resistance over surface hardness in clinical scenarios involving high occlusal loads, such as bruxism

This divergence underscores the anisotropic behavior of 3D-printed resins, where orientation-dependent interlayer bonding and layer alignment relative to applied loads influence mechanical outcomes differently.

## 4. Discussion

The present study evaluated the combined effects of build orientation and post-curing duration on the FS and VHN of a 3D-Printed Class IIa biocompatible occlusal splint resin. Both null hypotheses were rejected, confirming that manufacturing parameters significantly influence mechanical outcomes.

The 45° build orientation yielded the highest FS, while 90° orientation maximized VHN, reflecting the anisotropic nature of additively manufactured polymers. These results are consistent with previous findings showing that oblique orientations distribute stress more favorably, while vertical orientations enhance localized hardness but compromise fracture resistance [24]. Similarly, de Castro et al. [19] demonstrated that both build orientation and layer thickness significantly influenced the flexural strength of 3D-printed dental resins, while Kang et al. [20] reported that vertical orientation enhanced surface hardness but reduced dimensional accuracy, consistent with the anisotropic behavior observed in our study.

In the present study, the 45° build orientation produced the highest FS, whereas 90° orientation yielded the highest VHN. This appears inconsistent with reports where 0° or 90° orientations were superior in provisional or crown resins. Differences in resin formulation, layer thickness, and testing protocols may explain such discrepancies. One of the main reasons why conflicting results are reported in the literature regarding the superiority of different orientations is due to differences in the chemical structure of the resin formulations used. For example, methacrylate-based splint resins exhibit a higher degree of crosslinking, while dimethacrylate-based systems exhibit a more pronounced increase in hardness. Furthermore, variables in layer thickness and printer type directly influence the results. Differences in interlayer bonding have been reported between stereolithography (SLA), digital light processing (DLP), and LCD-based printing. Therefore, this suggests that the differences in the literature depend not only on the printing angle but also on the technology used. Several investigations have explored the influence of build orientation on the flexural and surface properties of 3D-printed dental materials, reporting variable outcomes depending on the resin system and testing methodology. Diken Turksayar et al. observed that 0° orientation produced the highest flexural strength in interim crowns [33], whereas Kim et al. found that both post-polymerization and print orientation significantly affected the biaxial flexural strength of splint resins [37]. Mudhaffer et al. reported that vertically printed specimens (90°) exhibited superior strength in permanent crown materials [38], while Derban et al. noted higher resistance at 0° for provisional resins [34].

In contrast, other studies have supported the mechanical advantages of oblique orientations. Alkhateeb et al. demonstrated that interim prostheses printed at 45° showed higher fracture resistance than those printed at 0° or 90° [39]. Similarly, Castro et al. observed peak microhardness values at this intermediate angle [19], suggesting improved polymer cross-linking and stress distribution. Çokakoğlu et al. further revealed that print orientation also influences the bonding strength of orthodontic brackets [40], highlighting how layer directionality can affect adhesion performance across different dental applications.

Collectively, these findings indicate that no single build angle is universally optimal, as outcomes are highly material- and test-dependent. For the resin tested here, 45° provided a balanced orientation that limited interlayer weaknesses observed at 90° and avoided the support-related flaws seen at 0°. In cases where high occlusal loads are applied, such as those caused by bruxism, fracture of the splint content (FS) is a critical observation. Indeed, patients with bruxism are subjected to repetitive high-load conditions, under which appliances with insufficient flexural strength are more prone to fracture or deformation. These findings emphasize selecting materials with high FS values to ensure clinical durability in such cases [41]. The influence of build orientation on flexural performance remains a debated issue. Casucci et al. [42] compared mechanical behavior across manufacturing technologies, noting material-dependent variability rather than orientation effects. Similarly, Mudhaffer et al. [38] found that both flexural strength and modulus were affected by orientation, and specimens printed at 45° exhibited higher resistance after artificial aging, confirming that the optimal build angle is material- and protocol-dependent. In contrast, Wulff et al. [32] reported that 90° orientation produced the highest biaxial flexural strength in occlusal splint disks. Such discrepancies may be attributed to differences in resin chemistry, testing methodology (three-point bending vs. biaxial flexure), and pre-conditioning protocols. In the present study, 45° orientation yielded the most favorable FS values, supporting the notion that the optimal build angle is highly material- and test-dependent.

Post-curing significantly improved both FS and VHN. Across orientations, cured specimens outperformed uncured ones, with FS increasing by 69–150% and VHN by up to 28%. Extended curing (20 min) further enhanced FS, while hardness gains showed smaller increments at longer durations, which is consistent with previous findings indicating a trend of reduced benefit beyond 40–60 min of post-curing [43]. The decision to employ a 20 min post-curing duration was based on the hypothesis that manufacturer recommendations often prioritize time efficiency over maximum mechanical properties. Doubling the exposure time serves as a pragmatic approach to identify the saturation point of polymerization, where significant cross-linking gains occur before the plateau effect reported in longer durations (e.g., >40 min) sets in [26,44]. Extending the post-curing time increases the cross-link density in the polymer chains, creating a more homogeneous structure. As a result, flexural strength increases significantly, while the increase in hardness plateaus after a certain point. This is because the surface layer is already highly polymerized, and additional light application is more effective in the interior. However, excessively long curing can also lead to increased brittleness and the risk of color stability deterioration. Therefore, the optimal time should be determined to enhance mechanical strength while not adversely affecting the material’s clinical use. Rosello Jimenez et al. [45] confirmed that aging processes can influence the mechanical stability and hardness of printable splint materials, supporting the plateau effect observed here. Likewise, Gad and Fouda [18] demonstrated that optimizing printing parameters, including post-curing time and layer thickness, can significantly affect the mechanical performance and flexural strength of 3D-printed dental resins.

In this study, a 45° build orientation combined with a 20 min curing cycle yielded the most favorable overall performance. While these findings are in vitro, other investigations have reported similar results. Casucci et al. [42] showed that a crown resin achieved its highest FS when printed at 45°, and Alkhateeb et al. [39] demonstrated superior fracture resistance of interim prostheses at this angle. de Castro et al. [19] confirmed that hardness can also peak at 45°. Regarding post-curing, Dantagnan et al. [44] reported that increasing the post-curing time and temperature significantly enhanced the degree of conversion of all tested resins, reaching its highest value at 90 min and 80 °C, although excessive heating led to a yellowish discoloration. Together, these findings support 45° with extended curing as a promising strategy to enhance splint durability. Nevertheless, Simoneti et al. [29] showed that microbial biofilm and surface property changes can alter the performance of interim crowns, emphasizing the need for caution when extrapolating in vitro data. Similarly, Ucar et al. [31] reported variability in mechanical properties among different denture base materials, reminding that material composition and clinical factors remain critical for outcome prediction. This guidance must be framed cautiously, since material composition, geometry, and intraoral factors may alter the optimal parameters, and long-term in vivo studies remain necessary. Although the present findings suggest that optimizing orientation and curing can substantially improve mechanical properties, clinicians should interpret these results with caution. Intraoral factors such as cyclic fatigue, humidity, enzymatic degradation, and occlusal dynamics may alter long-term performance, and further randomized clinical trials are warranted.

This study has several limitations. Only one resin–printer system was tested, limiting generalizability. The static ISO 4049 methods used do not replicate intraoral conditions, where cyclic fatigue, humidity, thermal changes, and biofilm can influence performance. Artificial aging protocols (e.g., thermal cycling, water storage, dynamic loading) were not included. Only FS and VHN were measured, whereas other properties such as fracture toughness, wear resistance, color stability, and residual monomer release are also clinically relevant. The 20 min curing time was chosen as a simple multiple of the manufacturer’s recommendation; other protocols (e.g., combined UV–thermal curing or nitrogen atmosphere) may yield different outcomes. Finally, clinical variables such as bruxism severity, occlusal load, and appliance thickness influence splint longevity and were not addressed here.

Future studies should therefore evaluate multiple resin–printer systems, incorporate artificial aging, and assess a broader range of mechanical and biological properties. Furthermore, parameters such as residual monomer release, cytotoxicity, and mucosal irritation are also important factors determining clinical success in terms of biocompatibility. Such biological parameters will contribute to more reliable clinical inferences beyond data obtained solely from mechanical testing. Ultimately, randomized clinical trials are required to confirm whether parameter optimization—such as 45° build orientation with 20 min curing—translates into improved long-term clinical outcomes.

## 5. Conclusions

Both build orientation and post-curing duration are critical manufacturing parameters that significantly influence the flexural strength, flexural modulus and Vickers hardness number of a 3D-printed occlusal splint resin. Flexural strength and modulus were maximized with a 45° build orientation combined with extended post-curing. Vickers hardness was maximized with a 90° orientation and extended curing time; however, this configuration produced the lowest flexural strength and modulus among the cured group. A balance must be struck between flexural strength and surface hardness; for high-load applications like occlusal splints, prioritizing flexural strength and modulus are clinically more important than maximizing hardness due to anisotropic material behavior.

## Figures and Tables

**Table 1:** Comparison of mean values of the flexural strength (MPa) of different curing times in different orientation.

Orientation	Curing Time	Flexural Strength		*p* *
		Mean	Standard Deviation	
0°	No Curing	65.06 ^A^	8.15	<0.001
	Standard Curing	149.40 ^B^	19.76	
	2× Curing	152.50 ^B^	15.58	
45°	No Curing	88.98 ^C^	8.33	<0.001
	Standard Curing	150.11 ^D^	6.49	
	2× Curing	169.76 ^E^	4.15	
90°	No Curing	55.70 ^F^	5.08	<0.001
	Standard Curing	128.50 ^G^	6.65	
	2× Curing	139.00 ^H^	2.47	

* Results of one-way analysis of variance test, Groups with different uppercase letters are significantly different (Tukey HSD test, *p* < 0.05).

**Table 2:** Comparison of mean values of the flexural modulus (MPa) of different curing times in different orientation.

Orientation	Curing Time	Flexural Modulus		*p* *
		Mean	Standard Deviation	
0°	No Curing	2196.17 ^A^	810.41	<0.001
	Standard Curing	5673.89 ^B^	860.93	
	2× Curing	6325.65 ^B^	659.73	
45°	No Curing	4110.38 ^C^	542.54	<0.001
	Standard Curing	4990.23 ^C^	1775.18	
	2× Curing	7502.17 ^D^	274.74	
90°	No Curing	2035.45 ^E^	390.78	<0.001
	Standard Curing	4749.91 ^F^	655.77	
	2× Curing	5392.17 ^G^	191.63	

* Results of one-way analysis of variance test, Groups with different uppercase letters are significantly different (Tukey HSD test, *p* < 0.05).

**Table 3:** Comparison of mean values of the Vickers Hardness (VHN) of different curing times in different orientations.

Orientation	Curing Time	Vickers Hardness		*p* *
		Mean	Standard Deviation	
0°	No Curing	17.39 ^A^	0.87	<0.001
	Standard Curing	22.00 ^B^	2.40	
	2× Curing	21.16 ^B^	1.51	
45°	No Curing	17.49 ^C^	0.90	<0.001
	Standard Curing	20.35 ^D^	1.57	
	2× Curing	19.37 ^D^	1.15	
90°	No Curing	17.07 ^E^	1.12	<0.001
	Standard Curing	21.40 ^F^	1.88	
	2× Curing	21.88 ^F^	1.75	

* Results of one-way analysis of variance test, Groups with different uppercase letters are significantly different (Tukey HSD test, *p* < 0.05).

## Data Availability

The datasets used and/or analyzed during the current study are available from the corresponding author on reasonable request.
